# The relationship between healthy lifestyles and bone health: A narrative review

**DOI:** 10.1097/MD.0000000000024684

**Published:** 2021-02-26

**Authors:** Bin Sheng, Xin Li, Andreas K. Nussler, Sheng Zhu

**Affiliations:** a The First Affiliated Hospital of Hunan Normal University, Hunan Provincial People's Hospital, Changsha, PR China; b Siegfried Weller Institute for Trauma Research, Eberhard Karls University Tuebingen, Department of Trauma and Reconstructive Surgery, BG Trauma Center Tuebingen, Tuebingen, Germany.

**Keywords:** bone health, cigarette smoke, lifestyle, nutrition, osteoporosis

## Abstract

**Background:**

Bone health, especially osteoporosis among ageing populations, has become an important topic for both clinical and basic researchers. The relationship between bone health and healthy lifestyles has been frequently discussed. The present study focuses on the relationship between bone health and healthy lifestyles among older adults, based on a global comparison.

**Methods:**

This narrative review was performed by collecting clinical trials, basic research and reviews on lifestyle and bone health in PubMed database.

**Results:**

Positive effects of physical activity and negative effects of malnutrition, alcohol abuse, and cigarette smoking on bone health were revealed. The relationship between bone health and drinking coffee and tea is still inconclusive. Moreover, the diversity of each region should be aware when considering healthy lifestyles to improve bone health.

**Conclusion:**

Healthy lifestyles are highly related to bone health, and different lifestyles may have different influences on regions with a high risk of bone diseases. It is practical to acknowledge the diversity of economic, religious, environmental and geological conditions in each region when providing suitable and effective recommendations for healthy lifestyles that can improve overall bone health.

## 1 Introduction

Insights into the biology and pathology of the human skeletal system have rapidly evolved over the last 3 decades. However, many aspects have yet to be fully clarified.^[1]^ Meanwhile, bone health, especially osteoporosis among ageing populations, has become an important topic for both clinical and basic researchers. In fact, osteoporosis is the primary reason for 8.9 million fractures per year globally.^[2,3]^ Currently, the treatment of osteoporosis is not completely effective, which may result in significant increases in medical insurance expenditures.^[4]^ Hence, it is important to determine the optimal way to prevent skeletal diseases, by having better awareness and creating the socioeconomic foundation for managing a healthy lifestyle.

Interestingly, the relationship between bone health and healthy lifestyles has been frequently discussed by both doctors and laymen, with a multitude of findings indicating which aspects are either good or bad for bone health. For example, many studies have acknowledged that long-term physical activity, a balanced diet and smoking cessation can have a positive influence on bone health. However, several questions are raised: Is it sufficient to simply respect the general guidelines? How can one manage a healthy lifestyle, which can be so distinctive and personal? Are all lifestyles consistent throughout the world? In this regard, the present study focuses on the relationship between bone health and healthy lifestyles based on a global comparison.

## 2 Methods

This narrative review was performed by collecting clinical trials, basic research and reviews on lifestyle and bone health. Articles that published in peer-reviewed scientific journals were included. Articles were excluded if they are not written in English language or published in peer-reviewed scientific journals.

### 2.1 Search strategy

We researched the PubMed database using keywords or combination of keywords.

“lifestyle,” “bone health,” “osteoporosis,” “physical activity,” “nutrition,” “dietary,” “calcium,” “vitamin,” “alcohol,” “coffee,” “tea,” and “cigarette smoking”. Articles published between January 1, 2003 and October 31, 2020 were selected. We further screened articles from peer-reviewed scientific journals that were written in English. All case reports were excluded.

### 2.2 Study selection

We screened for relevant articles, and selected articles for further reading based on the title and abstract. We read the articles that potentially fit our topic and all included articles were carefully discussed in the present review. Two thousand seven hundred eleven articles were included from the initial search, and 79 articles were selected and discussed in the review in the end. This narrative review does not need ethical approval, because no human/patient's data was used.

## 3 Results

### 3.1 Physical activity and exercise

Physical activity and exercise is generally recommended for improving overall health and mitigating a wide array of diseases, including coronary heart disease, certain types of cancer, Type-2 diabetes, metabolic syndrome, stroke and depression.^[5]^ Exercise is also the main physiological stimulus for both bone formation and metabolism.^[6]^ However, several questions are raised: Is the same exercise pattern suitable for all age groups? Is more exercise directly related to better bone health? Is light, moderate or vigorous exercise better for bone health?

Proverbially, human bone mineral density (BMD) incrementally increases during the first 3 decades of one's life, after which it starts to decline. Thus, exercise may have an impact on one's skeletal system during this period.^[7]^ In fact, previous studies on children and adolescents have demonstrated that physical exercise is significantly associated with both higher BMD and better microarchitecture of bone. However, studies on the bone effects of physical activity among older adults and the elderly people have found limited effects on BMD.^[8]^

As for children and adolescents, engaging in moderate or vigorous physical activity is associated with greater gains in bone mass,^[9]^ which is in line with the results of related studies of adolescents in Brazil^[10]^ and schoolchildren in Japan.^[11]^ Moreover, a systematic review found that weight-bearing activities, such as gymnastics and soccer, are related to better results in bone geometry, whereas non-weight-bearing exercises, such as swimming, do not have such an influence.^[12]^ In other studies, nine years of daily school-based exercise is associated with greater musculoskeletal gains, a significant reduction in fracture risk^[13]^ and longer duration of physical activity later in life.^[14]^

Regarding older adults and the elderly, it is conventionally considered that they should avoid vigorous physical activity. However, this belief is not definitive. For example, 1 study showed that females with more than 5 minutes of moderate to vigorous physical activity per day had higher trabecular bone scores, total hip T-scores and femoral neck T-scores than those with less than 5 minutes of such activity per day. Similarly, males with more than 20 minutes of moderate to vigorous physical activity per day had higher trabecular bone scores, total hip T-scores and femoral neck T-scores than those with less than 20 minutes of such activity per day.^[15]^ Moreover, among females 50 years and older, the substitution of 30 minutes of sedentary time with physical activity can increase BMD by approximately 3 mg/cm2 and reduce the risk of osteoporosis in the spine by roughly 12%.^[16]^ Based on the aforementioned findings, it is important to be aware that physical activity and exercise can be a powerful stimulus for bone formation among children and adolescents and for bone metabolism among older adults.^[17]^

**Figure 1:**
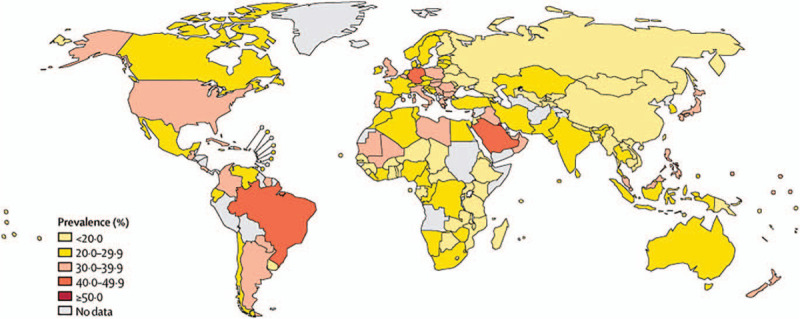
The prevalence of insufficient physical activity among males in 2016 (This is an Open Access article published under the CC BY 3.0 IGO license which permits unrestricted use, distribution, and reproduction in any medium).^[19]^

**Figure 2:**
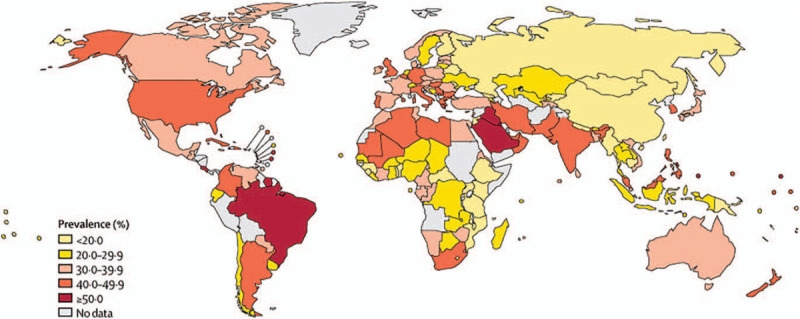
The prevalence of insufficient physical activity among females in 2016 (This is an Open Access article published under the CC BY 3.0 IGO license which permits unrestricted use, distribution, and reproduction in any medium).^[19]^

**Figure 3:**
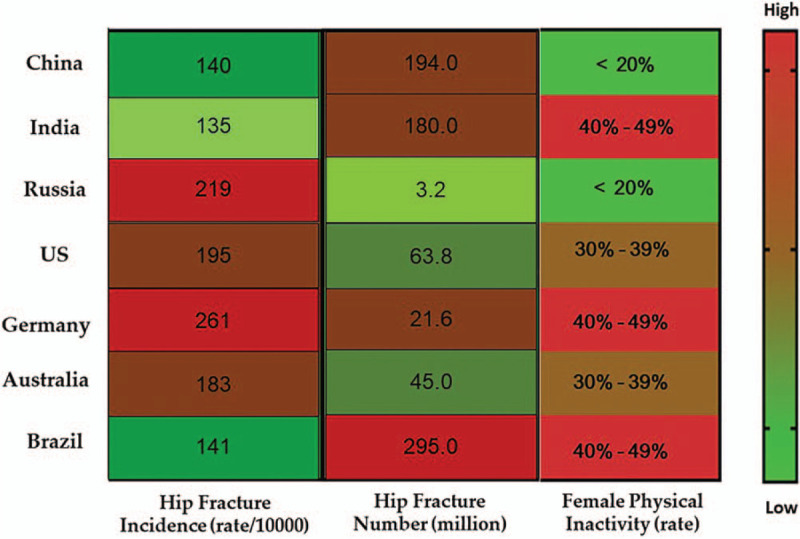
The prevalence of female physical inactivity among the representative countries with a high risk of osteoporosis.

As mentioned earlier, it is meaningful to determine the regional differences in physical activity around the world. According to the latest survey from Lancet Global Health (Fig. 1 and Fig. 2), the prevalence of physical inactivity among females is much higher than that of males. In terms of country diversity, high-income countries, such as the United States, several European countries and Japan, have a higher prevalence of physical inactivity than low-income countries, which is possibly due to more sedentary occupations and personal motorized transportation. Other high-prevalence groups are found in countries undergoing rapid urbanization, such as Brazil, Argentina and the Caribbean, which may be ignoring the importance of creating public open spaces and parks for physical activities (Fig. 3).^[18]^ Thus, greater awareness regarding the positive effects of physical activity and exercise should be made in these regions.

### 3.2 Nutrition and dietary patterns

Since food is generally the first necessity of humans, the relationship between food intake and health has been extensively explored. It is also widely known that malnutrition compromises almost every human organ as well as the skeletal system. In addition, the lack of essential substances for bone metabolism (e.g., protein, calcium and vitamins) may lead to bone dysplasia among children and delayed recovery for orthopedic patients.^[19]^ Conversely, obesity and overweight are widely known as strong risk factors for osteoporotic fractures, especially among the elderly.^[20]^

The supplementation of calcium and vitamin D is used in the prevention and treatment of almost all patients with osteoporosis. Calcium and vitamin D are recommended for people at risk of osteoporotic fracture.^[21]^ Diary consumption is a well-accepted calcium-supplementary strategy in reducing the risk of osteoporosis, but an umbrella review found no direct evidence that a high intake of dairy products may prevent osteoporotic fractures.^[22]^ A recent Bayesian network meta-analysis and meta-regression demonstrated that vitamin D supplementation does not affect risk of fractures, while supplementation with vitamin D together with calcium may have a beneficial effect for fracture prevention.^[23]^ High concentrations of maternal vitamin D may increase the bone mineral density for adults,^[24]^ the vitamin D dose needs to be further confirmed. Vegetables and fruits are considered major natural sources of vitamins, which may be beneficial for bone health.^[25]^ A latest umbrella review demonstrated a potential association between the intake of vegetables and reduced risk of hip fractures, but no evidence for the fruit intake.^[26]^ However, an increasing number of studies are proving that fruits have a positive effect on bone health because of their antioxidative contents.^[27]^ Overall, many supplementations are widely recognized and used for bone health benefits, but more research is needed to confirm the optimal form and dosage of dietary supplements, as well as their potential hazards.

As for children and adolescents, the findings of various studies have greatly differed. For example, a Korean study showed that obesity and overweight play a positive role in bone health in adolescents,^[28]^ which is in line with several studies.^[29]^ In contrast, an Indian and Chinese study claimed that overweight and obese children had less bone mass than their normal weight counterparts,^[30]^ which is similar to the results of related studies.^[31]^ The reason for such contradictions may be due to different sample sizes, regional influences and study designs. Nevertheless, malnutrition should be avoided during one's lifetime and weight should be emphatically controlled after puberty.

**Figure 4:**
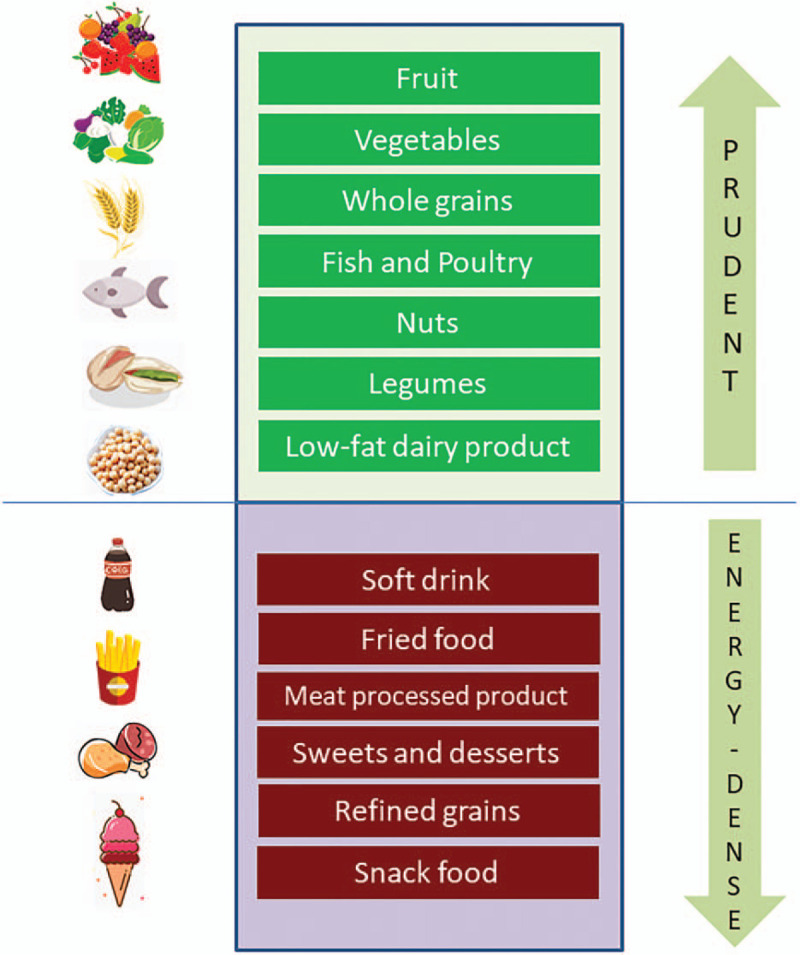
Two typical dietary patterns classified by food category.

It is important to note that one's dietary requirement goes well beyond just avoiding malnutrition in order to improve the quality of life. In this regard, nutrition and dietary patterns have proven to be controllable and modifiable tools for preventing metabolic bone disorders and osteoporosis.^[32]^ Although dietary patterns can be described in various ways,^[33]^ 2 patterns are generally recognized: the prudent dietary pattern (characterized by intakes of fruits, vegetables, whole grains, fish and poultry, nuts, legumes and low-fat dairy products); and the energy-dense dietary pattern characterized by intakes of foods with poor nutritional value such as sugar-sweetened beverages, processed meat products and snacks foods (Fig. 4).

Although numerous studies have highlighted the beneficial effects of the prudent dietary pattern on bone-related outcomes,^[34]^ a study of 10,991 participants (including a 20-year follow-up) showed that neither the prudent nor the energy-dense dietary pattern was associated with a greater risk of hip fractures in postmenopausal females or males over 50 years of age.^[35]^ Similarly, a Canadian study found no consistent relationship between diet and BMD.^[36]^ However, a recent literature review evaluated various studies on the relationship between dietary patterns and bone health and found that a bone-benefiting dietary pattern should emphasize the intake of fruits, vegetables, whole grains, fish and poultry, nuts, legumes and low-fat dairy products, but not soft drinks, fried foods, processed meats, etc.^[37]^ Mediterranean diet, emphasizing the intake of vegetables, fruits, herbs, nuts, legumes and whole grains, is proven to improve BMD and reduce the risk of fracture.^[38,39]^ Overall, despite some contrary results, the prudent dietary pattern is widely considered advantageous for higher BMD and lower fracture risk, especially among older adults.

**Figure 5:**
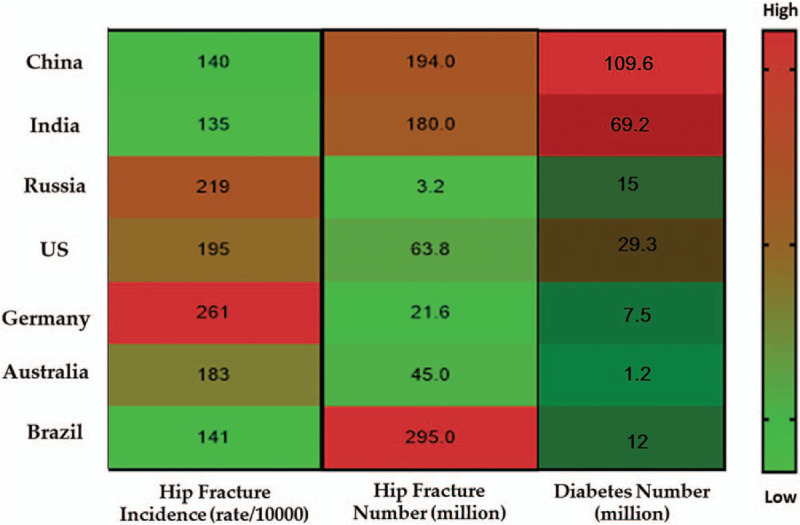
The number of diabetes among the representative countries with a high risk of osteoporosis.

With the rapid globalization of the world economy, the dietary habits of general populations are also changing. For instance, the global numbers of diabetes can be the representative parameters for examining the regional diversity of dietary patterns.^[40]^ Additionally, diabetes itself has been recently recognized as having significant connections with bone health and osteoporosis.^[41]^ Thus, it is undoubtedly instructive to determine the relationship between diabetes and osteoporosis among the countries mentioned earlier (Fig. 5). In this regard, since China, India, and the United States account for approximately 10% of the total number of diabetes patients in the world,^[42]^ the disease must first be dealt with on a national scale.

### 3.3 Drinking habits

Drinking beverages, such as alcohol, coffee, and tea, is an important non-essential component of daily diets around the world. For example, alcohol is regularly (and sometimes too frequently) consumed around the world, coffee is popular in Europe and the United States and tea is preferred by Asians. However, researchers and the general public are starting to pay attention to the optimal drinking habits that are most beneficial for one's health.

As for the consumption of alcohol, it contributes 5% to 10% of the daily energy requirements in many countries. More specifically, alcohol is first metabolized into acetaldehyde and then into acetate, which is important for the metabolism of energy, the maintenance of gut health and the regulation of appetite. However, excessive production of acetate can lead to the inhibition of gluconeogenesis and fatty acid oxidation.^[43]^ Hence, the consumption of alcohol can influence organ systems via its impact on nutrition or through the bioactivity of ethanol and its metabolites. Alcohol consumption is also related to skeletal health, fracture risk and osteoporosis, especially heavy drinking.^[44]^ Meanwhile, the definition of low, moderate and heavy drinking has differed among various studies. For example, according to the U.S. standard, moderate alcohol consumption is defined as 14 grams of pure ethanol per day, which is roughly equivalent to 12 Fluid Ounce (fl. oz). (340.92 ml.) of beer, 8 fl. oz. (227.28 ml.) of malt liquor, 5 fl. oz. (142.05 ml.) of wine or 1.5 fl. oz. (42.61 ml.) of 80-proof distilled spirits.^[45]^

**Figure 6:**
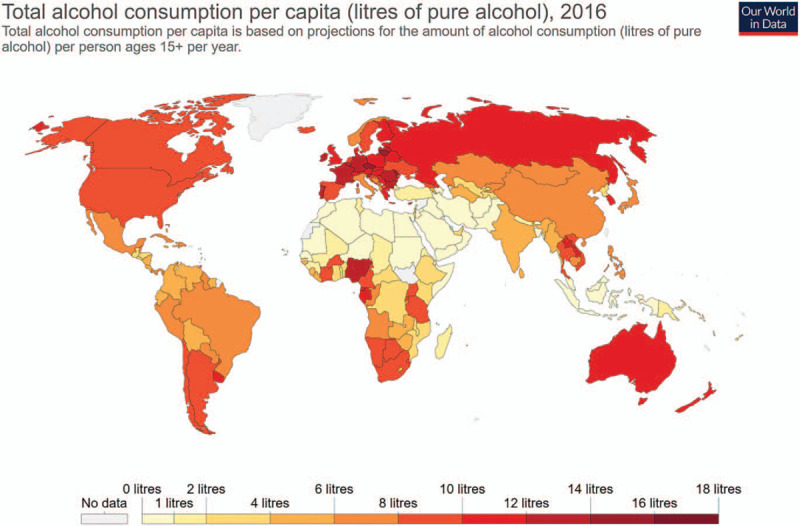
Total alcohol consumption per capita in 2016 (Source: Hannah Ritchie (2018) – “Alcohol Consumption”. Retrieved from: https://ourworldindata.org/alcohol-consumption’).

Some studies have also suggested that alcohol can acutely reduce the activity of both osteoblasts and osteoclasts,^[46]^ which may be the reason why the effects of alcohol on the skeletal system are influenced by drinking patterns. Interestingly, for moderate alcohol drinkers, alcohol consumption has been associated with higher BMD, lower fracture risk ^[47]^ and increased bone turnover markers.^[48]^ In contrast to moderate drinking, which appears to have potentially beneficial effects, chronic and/or heavy alcohol consumption is connected with decreased BMD and higher fracture risk.^[49]^ Moreover, a recent systematic review indicated that chronic drinkers (i.e., more than 2 drinks per day) can have 1.63 times the risk of osteoporosis.^[44]^ As for the world's total alcohol consumption per capita (Fig. 6), most African and western Asian countries are at the lower end of the scale, possibly due to economic and religious reasons, whereas North America and Europe are at the upper end of the scale. In fact, alcohol consumption across Eastern Europe, especially in Belarus, the Czech Republic and Lithuania is the highest at 14 to 17 liters per person per year. Thus, it is worth limiting alcohol consumption in these countries, especially for its potential benefit on bone health.

In sum, the 4 major mechanisms regarding heavy alcohol consumption and its effect on bone health are as follows. First, there is the nutrition level. As stated earlier, malnutrition is an indispensable risk factor for low BMD and chronic alcohol abuse is associated with malnutrition.^[50]^ In a related animal study, heavy alcohol consumption suppressed food consumption and weight gain.^[45]^ Second, there is the cellular level. In this case, numerous studies have shown the direct effects of ethanol on bone cells,^[45]^ while other investigations have revealed that alcohol can affect osteoblast activity.^[51]^ Third, there is the hormonal level. In this regard, inadequate 1, 25-dihydroxyvitamin D levels can negatively affect bone metabolism, due to impaired intestinal calcium absorption.^[52]^ Acute alcohol consumption can lead to transitory hypoparathyroidism, which is related to transient hypocalcaemia, hypercalciuria and hypermagnesuria.^[53]^ It can also impair the serotonergic-stimulatory regulation of growth hormone secretion and the growth hormone response to insulin-induced hypoglycaemia, which plays a crucial role in bone acquisition and remodeling.^[54]^ Moreover, heavy alcohol consumption can inhibit estrogen (particularly estrogen receptor isoforms Receptor α and Estrogen Receptor β), which has been shown to prevent bone loss.^[55]^ Finally, there is the signaling pathway level. In this case, chronic alcohol consumption can accelerate reactive oxygen species generation through increased expression of the NADPH oxidase (nicotinamide adenine dinucleotide phosphate oxidase oxidase) in osteoblasts, which is associated with bone resorption. Mitogen-activated Protein kinase signaling and the signal-regulated kinase pathways of Extracellular Regulated Protein Kinases, Nuclear Factor Kappa-B, c-Jun N-terminal Kinase, and Wingless-related integration site are also important for increasing nicotinamide adenine dinucleotide phosphate oxidase oxidase activity in alcohol-induced bone loss.^[56]^ However, there is still a lack of information regarding the signaling role of nicotinamide adenine dinucleotide phosphate oxidase oxidase enzymes in osteoclastic bone resorption and osteoclastogenesis.^[57]^

In regard to coffee, it is prevalent throughout the world, especially in Western countries. Although it has been associated with different health benefits,^[58]^ there are inconsistencies in the correlation between coffee consumption and bone health. For instance, recent evidence demonstrated that moderate coffee intake is not significantly associated with risk of hip fractures and fruiters, however an increasing trend is shown for hip fractures in men.^[59,60]^ No significant association between coffee consumption and musculoskeletal outcomes was found in developed countries.^[61]^ Interestingly, a recent meta-analysis suggested that daily consumption of coffee increases the risk of fractures in females, but decreases such risk in males.^[62]^ Moreover, a Taiwanese study found that coffee drinking is associated with a lower risk of osteoporosis in males and premenopausal females.^[63]^ Conversely, a Chinese study showed that drinking at least 4 cups of coffee per day is associated with a higher hip fracture risk, while moderate coffee consumption may alleviate such risk in postmenopausal females.^[64]^ Based on these findings, the relationship between coffee consumption and bone health is still inconclusive.

Finally, as one of the most popular beverages in the world, tea has been reported to prevent cardiovascular diseases, rheumatoid arthritis, influenza and cancer, although some studies have yielded inconsistent conclusions regarding the effect of tea on bone health.^[65,66]^ However, the latest meta-analyses showed that habitual tea consumption is positively associated with higher BMD,^[65]^ regardless of the type of tea.^[67]^ Moreover, the flavonoid that exists in all types of tea has been widely reported to have positive effects on bone metabolism. On the one hand, the flavonoid is positively correlated to antioxidant status, which can down-regulate oxidative stress and prevent bone resorption.^[68]^ On the other hand, the flavonoid can react to osteoblasts by increasing mineralization and alkaline phosphatase activity^[69]^ as well as mimicking the ability of estrogen to positively influence bone turnover.^[70]^

### 3.4 Smoking habits

There are approximately 130 million long-term smokers in the world, resulting in roughly 6 million deaths per year, with passive smoking causing more than 600,000 deaths.^[71]^ Although the deleterious effects of cigarette smoke on the musculoskeletal system have been attested by numerous studies,^[71]^ it is important to discuss whether smoking is harmful to bone health. To date, the relationship between smoking cessation and bone health remains inconclusive. For example, some studies have shown that only postoperative patients are strongly suggested to stop smoking during hospitalization, since it prevents wound healing.^[26]^ Meanwhile, a prospective study of postmenopausal females showed that 6 weeks of smoking cessation help reduce sex hormone-binding globulin and N-terminal collagen cross-links,^[72]^ while another study indicated that smoking cessation is associated with increased body weight, lean mass, appendicular skeletal muscle index, fat mass, bone mineral content, BMD and muscle strength. Conversely, another study found no significant reductions in BMD among continuing smokers.^[73]^ Based on these findings, it is apparent that further studies are necessary.

**Figure 7:**
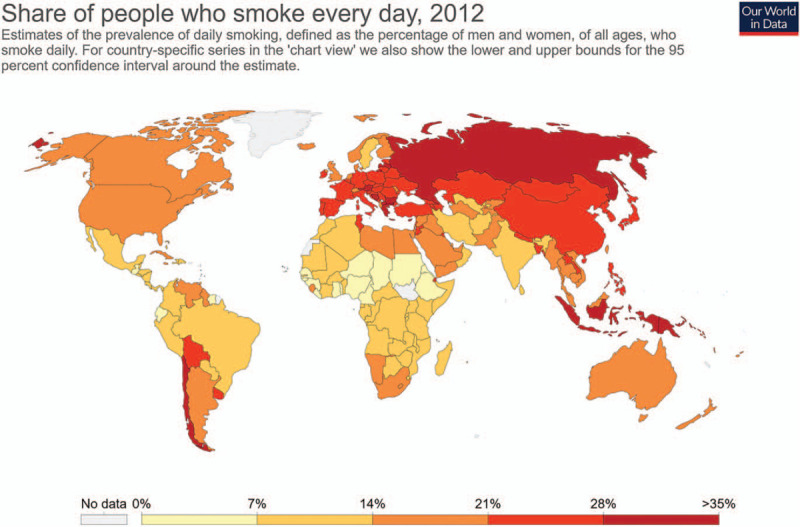
The share of people who smoke every day in 2012 (Source: Hannah Ritchie and Max Roser (2013) – “Smoking”. Published online at OurWorldInData.org. Retrieved from: ’https://ourworldindata.org/smoking’).

As for the share of long-term smokers in the world (Fig. 7), Russia has the highest percentage, followed by China and Europe. Meanwhile, North America and Australia have a moderate share of smokers. Since continuous smoking has proven to be harmful for bone health, it is important to emphasize the negative effects of smoking and control the market in these regions. Conversely, other countries with a relatively low share of smokers, such as India and Brazil, might not need to perform such actions.

Moreover, E-cigarettes, also known as electronic nicotine delivery systems, have become the new trend in the recent decade. They are not only claimed to be a safer and healthier alternative to conventional tobacco products, but they are also marketed as a smoking cessation tool. Emerging on the Chinese market in 2004 and the United States in 2007, E-cigarettes have become a multi-billion dollar industry.^[74]^ Some researchers have suggested a decrease in the disease burden of E-cigarettes, compared to combustible cigarettes.^[75]^ However, E-cigarette liquids may be cytotoxic, due to their aerosol emissions, which have been shown to exert negative effects on animals.^[76]^ Thus, determining whether E-cigarettes help reduce tobacco smoking or minimise the harm for individuals who cannot (or will not) quit smoking conventional cigarettes has been the subject of increasing debates.^[77]^ Meanwhile, studies have produced varying results. For instance, one study showed that E-cigarettes decrease the proliferation of mesenchymal cells,^[78]^ while another study showed that the flavoured E-liquids reveal collagen Type-1 structural elements that can elucidate the potential consequences of E-cigarette use in bone.^[78]^ In short, with the proliferation of E-cigarette use and increasing expenditures, especially among teenagers, high-quality studies are necessary.

## 4 Conclusion

**Figure 8:**
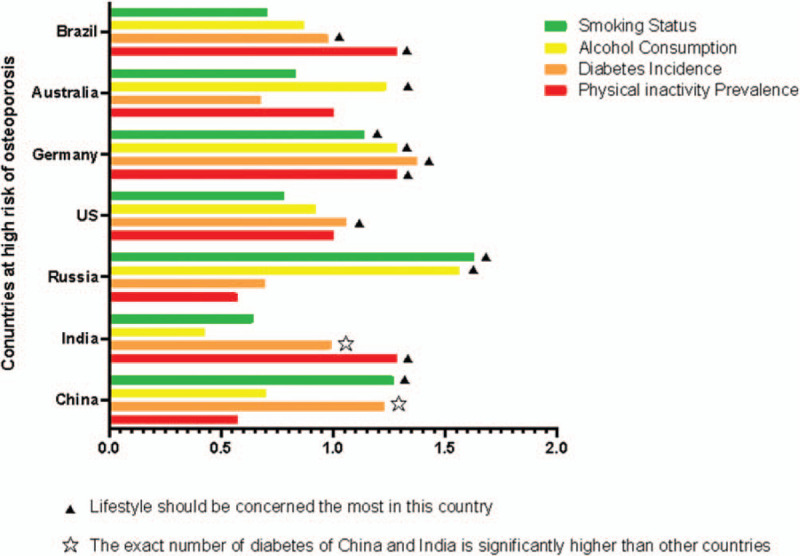
The influence of major lifestyles in the representative countries with a high risk of osteoporosis.

The present study examined the relationship between bone health and healthy lifestyles based on a global comparison. According to the findings, healthy lifestyles are highly related to bone health, especially BMD and the risk of fracture. In terms of the human skeletal system, life-long moderate to vigorous physical activity, the prudent eating pattern, moderate beverage consumption and no tobacco smoking are highly recommended. Moreover, this study identified the representative countries in the world with a high risk of osteoporosis and discovered that different lifestyles may have different influences on these regions (Fig. 8). For example, physical activity should be the priority in Brazil, Germany, and China, due to their relatively high instances of diabetes. Meanwhile, since the rate of smoking and alcohol consumption in Russia is the highest in the world, greater awareness should be raised among the general population regarding the negative effects of such actions. Finally, since each region includes a multitude of factors, such as economic, religious, environmental and geological conditions, it is practical to acknowledge the diversity of each region when providing suitable and effective recommendations for healthy lifestyles that can improve overall bone health.

## Acknowledgments

We would like to thank the First Affiliated Hospital of Hunan Normal University (Hunan Provincial People's Hospital) for promoting the international cooperation.

## Author contributions

**Conception:** Bin Sheng, Sheng Zhu.

**Supervision:** Andreas K. Nussler, Sheng Zhu.

**Visualization:** Xin Li, Sheng Zhu.

**Writing – review & editing:** Andreas K., Nussler, Sheng Zhu.

**Writing – original draft:** Bin Sheng, Xin Li, Sheng Zhu.
